# Genome-wide end-sequenced BAC resources for the NOD/MrkTac^☆^ and NOD/ShiLtJ^☆☆^ mouse genomes

**DOI:** 10.1016/j.ygeno.2009.10.004

**Published:** 2010-02

**Authors:** Charles A. Steward, Sean Humphray, Bob Plumb, Matthew C. Jones, Michael A. Quail, Stephen Rice, Tony Cox, Rob Davies, James Bonfield, Thomas M. Keane, Michael Nefedov, Pieter J. de Jong, Paul Lyons, Linda Wicker, John Todd, Yoshihide Hayashizaki, Omid Gulban, Jayne Danska, Jen Harrow, Tim Hubbard, Jane Rogers, David J. Adams

**Affiliations:** a The Wellcome Trust Sanger Institute, Hinxton, UK; b The Genome Analysis Centre, Norwich Research Park, Norwich, UK; c Children's Hospital Oakland Research Institute, Oakland, CA, USA; d Cambridge Institute for Medical Research, Cambridge, UK; e RIKEN, Yokohama Institute, Japan; f Hospital for Sick Children Research Institute and University of Toronto, Toronto, Canada

**Keywords:** Bacterial artificial chromosome, NOD/MrkTac, NOD/ShiLtJ, Mouse genome, Non-obese diabetic (NOD), Type 1 diabetes, T1D, Insulin-dependent diabetes, IDD

## Abstract

Non-obese diabetic (NOD) mice spontaneously develop type 1 diabetes (T1D) due to the progressive loss of insulin-secreting β-cells by an autoimmune driven process. NOD mice represent a valuable tool for studying the genetics of T1D and for evaluating therapeutic interventions. Here we describe the development and characterization by end-sequencing of bacterial artificial chromosome (BAC) libraries derived from NOD/MrkTac (DIL NOD) and NOD/ShiLtJ (CHORI-29), two commonly used NOD substrains. The DIL NOD library is composed of 196,032 BACs and the CHORI-29 library is composed of 110,976 BACs. The average depth of genome coverage of the DIL NOD library, estimated from mapping the BAC end-sequences to the reference mouse genome sequence, was 7.1-fold across the autosomes and 6.6-fold across the X chromosome. Clones from this library have an average insert size of 150 kb and map to over 95.6% of the reference mouse genome assembly (NCBIm37), covering 98.8% of Ensembl mouse genes. By the same metric, the CHORI-29 library has an average depth over the autosomes of 5.0-fold and 2.8-fold coverage of the X chromosome, the reduced X chromosome coverage being due to the use of a male donor for this library. Clones from this library have an average insert size of 205 kb and map to 93.9% of the reference mouse genome assembly, covering 95.7% of Ensembl genes. We have identified and validated 191,841 single nucleotide polymorphisms (SNPs) for DIL NOD and 114,380 SNPs for CHORI-29. In total we generated 229,736,133 bp of sequence for the DIL NOD and 121,963,211 bp for the CHORI-29. These BAC libraries represent a powerful resource for functional studies, such as gene targeting in NOD embryonic stem (ES) cell lines, and for sequencing and mapping experiments.

## Introduction

Type 1 diabetes (T1D) or insulin-dependent diabetes (IDD) is a polygenic disorder, characterized by hyperglycaemia that results from the autoimmune T cell-mediated destruction of the insulin-producing β-cells of the islets of Langerhans of the pancreas [1–3]. T1D is triggered by different environmental and genetic factors and has variable penetrance, suggesting that susceptibility to this syndrome is inherited and polygenic [1–6].

The non-obese diabetic (NOD) mouse is an experimental model for human T1D, developed in Japan by Makino et al. [7]. NOD mice spontaneously develop T1D following an autoimmune mediated process that progressively destroys their insulin-secreting β-cells [2,3]. T1D is typically associated with allelic variants of the Human Leukocyte Antigen (HLA) class II immune response genes within the Major Histocompatability Complex (MHC) [3,8]. Genetic analysis of the NOD mouse has further established that the inheritance of diabetes in these mice is controlled polygenically by at least 27 disease-associated loci, distributed over at least 14 different chromosomes [9]. These loci have been designated *Idd* loci, for *insulin-dependent diabetes* [9,10].

Understanding how these loci contribute to the development of T1D in the NOD mouse should inform us of the underlying mechanisms of T1D development in humans. It is important, however, to analyse *Idd* loci in the context of the genome in which they reside so that the effect of the background in which the phenotype is observed, and the role of epistatic genetic interactions can be assessed. Bacterial artificial chromosomes (BACs) represent a useful resource for sequencing, mapping and functional studies [11]. Here we describe the development and end-sequencing of two BAC libraries for the NOD substrains NOD/MrkTac (DIL NOD) and NOD/ShiLtJ (CHORI-29). While NOD/MrkTac and NOD/ShiLtJ mice are derived from the same founding stock of NOD mice developed by intercrossing Jcl:ICR (Institute for Cancer Research) mice for more than 20 generations [7] they have been maintained as isolated colonies for many generations, and as such are likely to have diverged significantly. Indeed these NOD substrains show subtle differences in the timing and presentation of diabetes, and also in their plasma glucose levels. The availability of BAC libraries for both of these NOD substrains will allow us to study the differences between them and to gain a better understanding of the pathogenesis of T1D. In addition, with the recent advent of embryonic stem (ES) cells derived from NOD mice [12,13] these BAC libraries will form the foundation for targeted manipulation of the NOD mouse genome.

## Results

### End-sequencing

**Fig. 1:**
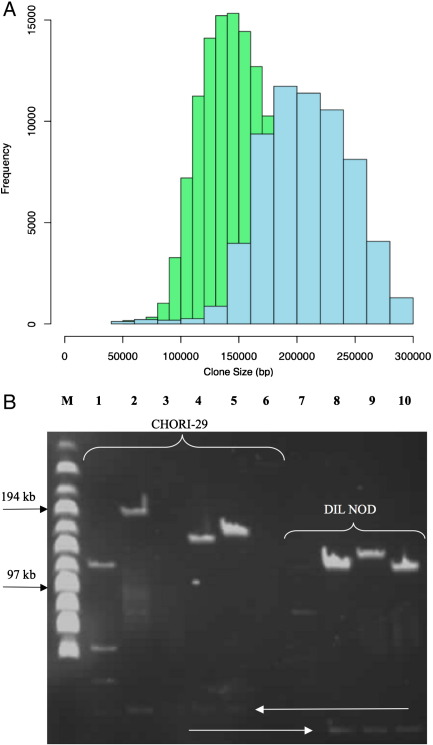
DIL NOD clones have an average insert size of 149,809 bp and CHORI-29 clones have an average insert size of 205,413 bp. (A) Frequency distribution of the insert size of the 125,266 DIL NOD clones (green) and the 62,162 CHORI-29 clones (blue). (B) CHEF gel of *Not*I digested clones showing the difference in size between the NOD libraries. The two different vector bands are illustrated with white arrows. Marker (M) is a DNA marker of 24–300 kb size range.

All clones from the DIL NOD and CHORI-29 BAC libraries were end-sequenced and the sequence read data have been submitted to EMBL. These data are also available from the Ensembl trace repository (http://trace.ensembl.org/) and the NCBI Trace Archive (http://www.ncbi.nlm.nih.gov/Traces/trace.cgi). 332,535 DIL NOD BAC clone end-sequences successfully passed post-sequencing quality processing from a total of 196,032 BACs, generating 229,736,133 bp of sequence. Of these passed reads, 318,065 (95.6%) were aligned to the C57BL/6J reference genome (NCBIm37), 170,029 (53.5%) of which were aligned to a single definitive location (Table 1A). Similarly for the CHORI-29 library, 170,159 BAC clone end-sequences passed post-sequencing quality processing from 110,976 BACs, generating 121,963,211 bp of sequence. Of these passed reads, 159,574 (93.8%) were aligned successfully to the reference C57BL/6J genome with 80,710 (50.6%) reads aligned to a single definitive location on NCBIm37 (Table 1B). The majority of the reads that did not map contained repetitive sequences or were of low quality. Both sets of data can be downloaded from the Sanger FTP site (ftp://ftp.sanger.ac.uk/pub/NODmouse/NOD_BACend_alignments). Mapping was performed using SSAHA2 with default parameters [14]. Using read-pair information we could place 41,468 DIL NOD clones and 18,257 CHORI-29 clones unambiguously on the genome since both read-pairs matched uniquely. However, it was also possible to establish the position of certain clones for which only one end mapped uniquely where the other end of the clone mapped to the genome within 3 standard deviations of the mean insert length of clones from the library and on the opposite sequence strand. This allowed us to place a further 83,796 DIL NOD clones and 43,905 CHORI-29 clones on the genome, resulting in a total of 125,266 uniquely placed DIL NOD clones and 62,162 uniquely placed CHORI-29 clones. The different success rates in the unique positioning of the BAC clones from these libraries to the genome was largely due to differences in the quality of end-sequence data produced from both libraries. CHORI-29 clones have larger genomic inserts and as a consequence were harder to prep and sequence compared to clones from the DIL NOD BAC library. Using the mapping data for both libraries it was possible to estimate the average insert size for the DIL NOD library to be 149,809 bp and 205,413 bp for the CHORI-29 library (Fig. 1A), which correlated with the experimentally derived figures (Fig. 1B).

### Physical genome coverage and coverage of Ensembl genes

The average depth of genome coverage of the end-sequenced DIL NOD library was calculated to be 7.1-fold across the autosomes and 6.6-fold across the X chromosome (Table 2A). The end-sequenced CHORI-29 library has an average depth of 5.0-fold and 2.8-fold across the autosomes and the X chromosome, respectively (Table 2B). The total number of Ensembl [15] predicted genes that are fully covered by a BAC clone from the DIL NOD library is 31,093 (98.8%) and 30,103 (95.7%) for the CHORI-29 library, based upon Ensembl mouse release 55 (NCBIm37). The total number of Ensembl genes partially covered by DIL NOD and CHORI-29 BACs is 349 (1.1%), and 1,351 (4.3%) respectively, 200 (0.6%) of which are partial in both libraries. The total number of Ensembl genes contained completely in DIL NOD and CHORI-29 BAC gaps is 30 (0.1%) and 18 (0.06%) respectively. There are 5 (0.02%) genes that are present on the reference genome but absent completely from both NOD libraries. This is likely because it was not possible to place one or both BAC end-reads of a pair due to reference genome gaps adjacent to these Ensembl genes. These data are available on the Sanger FTP site (ftp://ftp.sanger.ac.uk/pub/NODmouse/NOD_Ensembl_gene_coverage).

**Fig. 2:**
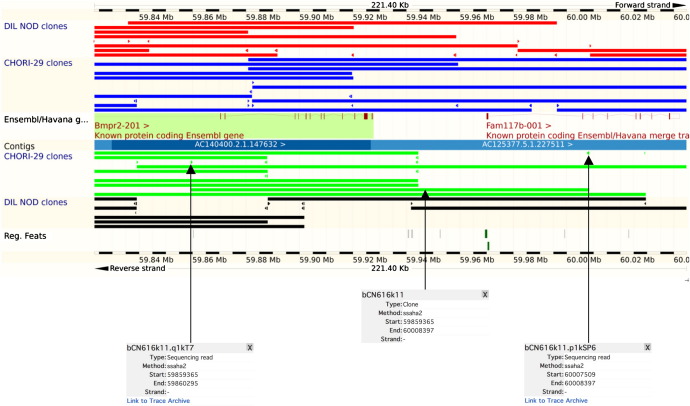
NOD BAC clones are displayed on the Ensembl genome browser under the DAS sources, http://www.ebi.ac.uk/das-srv/genomicdas/das/nod_clones_m37 and http://www.ebi.ac.uk/das-srv/genomicdas/das/chori29_clones_m37. The NOD/MrkTac mouse strain is designated DIL NOD (Sanger/Ensembl prefix bQ) and the NOD/ShiLtJ mouse strain is designated CHORI-29 (Sanger/Ensembl prefix bCN). The DIL NOD clones are displayed as black and red bars and the CHORI-29 clones are displayed as blue and green bars, the colours indicating the orientation of the DNA insert in the vector. BAC clones on the forward strand are drawn above the DNA contig while clones on the reverse strand are drawn below. The clone end-reads are shown as small arrows in the corresponding color to the relevant BAC clone. Links to the end-read sequences in the Ensembl trace archive can be found by clicking on the clone end of interest.

To make the data accessible to the wider community, we have generated a Distributed Annotation System (DAS) [16] source to display both the DIL NOD clones (http://www.ebi.ac.uk/das-srv/genomicdas/das/nod_clones_m37) and the CHORI-29 clones (http://www.ebi.ac.uk/das-srv/genomicdas/das/chori29_clones_m37) so that they can be visualized in DAS source compliant browsers. For example, in the Ensembl genome browser (http://www.ensembl.org/Mus_musculus/Info/Index) the alignments of both NOD BAC libraries can be accessed through the DAS sources menu and viewed against the reference C57BL/6J genome. DIL NOD clones are displayed as red and black lines depending on the orientation of the insert in the vector, while CHORI-29 clones are displayed as green and blue lines. The BAC end-sequences can also be viewed as traces in the main “Region in Detail” window of the Ensembl genome browser. The method used to display BAC ends in Ensembl shows only those that have corresponding ends that are considered to be within 3 standard deviations of the mean insert size of the library. End-reads provide a link to the Ensembl trace repository (http://trace.ensembl.org), where the end-read sequences for all quality clipped reads have been deposited (Fig. 2). FASTA files of these quality clipped reads have also been generated and deposited on the Sanger FTP site (ftp://ftp.sanger.ac.uk/pub/NODmouse/NOD_BACend_fasta_sequences).

### Analysis of nucleotide variation

We used SSAHA-SNP2 [15] to call single nucleotide polymorphisms (SNPs) and deletion insertion polymorphisms (DIPs) from both the DIL NOD and CHORI-29 end-sequence reads by comparing them to the NCBIm37 C57BL/6J assembly. We called 191,841 SNPs and 15,824 DIPs for DIL NOD and 114,380 SNPs and 4,942 DIPs for CHORI-29. These data are available on the Sanger FTP site (ftp://ftp.sanger.ac.uk/pub/NODmouse/NOD_variation_data). The following criteria were used: the identity had to be greater than or equal to 92% match length, greater than or equal to 80% of the read length and a match score or match length greater than 250 bp. These SNPs have been validated against Illumina whole genome shotgun data of the NOD/ShiLtJ genome (http://www.sanger.ac.uk/modelorgs/mousegenomes/) and submitted to dbSNP. However, it is important to note that the DIPs are candidate nucleotide variants and follow-up genotyping is warranted.

## Discussion

The genome-wide DIL NOD and CHORI-29 mouse BAC end-sequenced libraries provide a unique way of studying T1D in mouse. The two libraries were aligned against the C57BL/6J mouse genome and are displayed on the Ensembl genome browser, which almost eliminates the need to perform filter hybridizations to isolate clones of interest, except for “non-reference” regions of the genome that are novel in NOD mouse. The distribution of mapped sequence reads is relatively even across the genome, with the exception of the Y chromosome, which is not represented in the DIL NOD mouse library. To date, high-quality BAC libraries exist for several mouse strains, including C57BL/6J, MSM/Ms [17,18], C3H/HeJ, BALB/c, A/J, SPRET/Ei, AKR/J, CAST/Ei (http://bacpac.chori.org/home.htm) and 129S7 [11], with BAC end-sequences existing for C57BL/6J, MSM/Ms and 129S7. Such libraries have been shown to be an invaluable resource for assembling genomes and for *in vivo* functional studies, such as BAC rescue [19–21].

The high-density and end-sequence quality of these BAC libraries make them useful tools for examining large-scale structural differences between the two substrains of NOD and other mouse strains and will greatly facilitate high-throughput targeted manipulation of the NOD mouse genome. With the recent advent of NOD ES lines these BAC libraries will be a critical resource for targeting vector construction [12,13] where isogenic DNA has been shown to be critically important in obtaining high targeting frequencies [22]. Due to the high coverage of the C57BL/6J reference genome by the NOD BAC ends, regions with poor coverage of the reference genome may represent structural variants in the NOD mouse genome when compared to the reference genome. Importantly we are currently performing targeted sequencing of regions of the NOD mouse genome relevant to T1D, which is crucial for understanding the role genetic susceptibility plays in the pathogenesis of T1D (http://www.sanger.ac.uk/Projects/M_musculus-NOD/). We are also in the process of using the Illumina platform [23] to sequence the entire NOD/ShiLtJ genome, which will greatly improve the utility of these BAC resources, and should help to position unaligned BACs to novel ‘non-reference’ regions of the NOD genome (http://www.sanger.ac.uk/modelorgs/mousegenomes/).

## Materials and methods

### Construction of the BAC libraries

The DIL NOD BAC library was constructed from NOD/MrkTac female liver genomic DNA at the Genomic Sciences Center, RIKEN in collaboration with the Diabetes and Inflammation Laboratory at Cambridge University. *Eco*RI was used to partially digest whole genomic DNA and the resulting fragments were cloned in pBACe3.6 as described previously [18]. These clones are available from Dr Jayne Danska jayne.danska@sickkids.on.ca. The CHORI-29 library was constructed in a similar manner at the Children's Hospital, Oakland, California, USA. NOD/ShiLtJ male kidney genomic DNA, obtained from the Jackson Laboratory, was cloned in pTARBAC2.1 and these clones are available from http://bacpac.chori.org/. The average insert size was experimentally verified using clamped hexagonal electric field (CHEF) electrophoresis, a system similar to pulse field gel electrophoresis (PFGE) [24]. The marker (M) (Fig. 1B) was a MidRange II PFG Marker – N3552S from New England BioLabs. For storage purposes, the DIL NOD library was arrayed into 527 384-well plates and the CHORI-29 library was replicated into 672 384-well plates.

### End-sequence profiling of the BAC resource

In total 378,896 reads were attempted for the DIL NOD library using T7 and SP6, M13-21 and pUCR, and 3HPPSK and 3HPpur primers on the vector and big dye terminator chemistry. 207,321 reads were attempted for the CHORI-29 library using the T7 and SP6 primers on the vector, and big dye terminator chemistry. Sequence-reads were subjected to processing using Automated Sequence Preprocessing (ASP) [25]. The number of insertless clones was determined to be 3% for DIL NOD and 1% for CHORI-29. Average read-lengths were determined to be 694.08 bp in length for DIL NOD and 718.77 bp in length for CHORI-29.

### End-sequence mapping of BAC clones

End-read data were mapped using SSAHA2 with the mapping criteria that more than 100 bp should map with greater than 95% identity to the NCBIm37 assembly. Clone-ends were iteratively aligned against the genome and after each round the average size of the clones was calculated as well as the standard deviation. Clone-ends that were plus or minus three standard deviations away from the mean were rejected. Clones with only one end aligned, ends that were orientated in the same direction, or ends that lie at unrealistic distances were rejected.

## Conflict of interest statement

The authors declare that they have no conflict of interest.

## Figures and Tables

**Table 1A:** 

BAC clones in library	196,032
Attempted BAC clone end-reads	378,896
BAC clone end-reads sequenced successfully	332,535
Passed BAC clones sequenced on both strands	150,878
BAC clone end-reads with an alignment to the genome	318,065
Aligned BAC clone end-reads with a unique match	170,029
Aligned BAC clone end-reads with multiple matches	148,036
BAC clones positioned using unique end-read pairs	41,468
BAC clones positioned uniquely	125,266

Sequencing and alignment summary for the DIL NOD BAC library.

**Table 1B:** 

BAC clones in library	110,976
Attempted BAC clone end-reads	207,321
BAC clone end-reads sequenced successfully	170,159
Passed BAC clones sequenced on both strands	75,046
BAC clone end-reads with an alignment to the genome	159,574
Aligned BAC clone end-reads with a unique match	80,710
Aligned BAC clone end-reads with multiple matches	78,864
BAC clones positioned using unique end-read pairs	18,257
BAC clones positioned uniquely	62,162

Sequencing and alignment summary for the CHORI-29 BAC library.

**Table 2A:** 

Chr. name	Total chr. length bp	Non-redundant clone-length bp	Clone-length total bp	Passed aligned sequence bp	Clone depth	% total coverage
1	197,195,432	189,902,383	1,385,443,533	13,593,208	7.03	96.3
2	181,748,087	177,813,774	1,348,761,746	13,129,801	7.42	97.84
3	159,599,783	155,666,906	1,151,145,108	11,396,911	7.21	97.54
4	155,630,120	150,659,365	1,134,909,770	10,944,727	7.29	96.81
5	152,537,259	146,223,145	1,009,782,448	9,773,915	6.62	95.86
6	149,517,037	145,684,306	1,049,822,257	10,281,087	7.02	97.44
7	152,524,553	140,866,414	1,007,473,869	9,637,732	6.61	92.36
8	131,738,871	123,985,369	907,535,329	8,826,036	6.89	94.11
9	124,076,172	120,302,728	916,247,038	8,800,500	7.38	96.96
10	129,993,255	125,971,520	897,618,520	8,764,981	6.91	96.91
11	121,843,856	118,333,935	924,494,065	8,842,955	7.59	97.12
12	121,257,530	117,098,461	854,759,223	8,258,226	7.05	96.57
13	120,284,312	116,142,787	888,982,416	8,609,816	7.39	96.56
14	125,194,864	119,290,632	846,844,974	8,247,811	6.76	95.28
15	103,494,974	100,431,693	735,484,668	7,116,922	7.11	97.04
16	98,319,150	94,516,190	692,930,172	6,811,564	7.05	96.13
17	95,272,651	91,304,604	690,760,336	6,680,800	7.25	95.84
18	90,772,031	86,713,096	655,698,203	6,460,483	7.22	95.53
19	61,342,430	57,670,646	421,571,236	4,064,957	6.87	94.01
X	166,650,296	160,833,108	1,095,695,031	10,991,837	6.57	96.51
Y	15,902,555	0	0	0	0	0

All data in Table 1 and Table 2 are derived from the NCBIm37 mouse assembly. The DIL NOD library covers over 95.6% of the mouse genome at an average depth of 7.1-fold across the autosomes and 6.6-fold across the X chromosome. Column 2 is the C57BL/6J chromosome length. The non-redundant clone-length field (column 3) is the non-redundant BAC clone sequence estimated for that chromosome, using both read-pairs. This may count each base several times, the number of times being the number of BACs overlapping that position. The clone-length total field (column 4) is the total sequence estimated for all the BACs for that chromosome, using both read-pairs. The passed aligned sequence column (5) is the total number of sequenced bases per chromosome that have successfully passed quality control and been mapped. The clone depth field (column 6) is the clone-length total (column 4) divided by the total chromosome length (column 2). The % total coverage (column 7) is the non-redundant clone-length total (counting each base covered only once) as a percentage of the total chromosome length.

**Table 2B:** 

Chr. Name	Total chr. length bp	Non-redundant clone-length bp	Clone-length total bp	Passed aligned sequence bp	Clone depth	% total coverage
1	197,195,432	188,433,001	1,046,993,130	7,724,484	5.31	95.56
2	181,748,087	175,429,538	942,386,790	6,916,061	5.18	96.52
3	159,599,783	154,711,914	875,284,125	6,490,661	5.48	96.94
4	155,630,120	148,498,769	827,737,353	6,127,905	5.32	95.42
5	152,537,259	144,530,268	733,517,218	5,404,820	4.81	94.75
6	149,517,037	143,599,805	775,532,299	5,768,104	5.19	96.04
7	152,524,553	135,008,655	644,735,047	4,788,996	4.23	88.52
8	131,738,871	121,214,563	606,581,664	4,522,109	4.60	92.01
9	124,076,172	118,654,230	598,157,862	4,362,992	4.82	95.63
10	129,993,255	124,749,578	675,601,360	4,987,613	5.20	95.97
11	121,843,856	115,930,403	605,273,345	4,474,962	4.97	95.15
12	121,257,530	115,274,597	604,649,354	4,482,487	4.98	95.07
13	120,284,312	114,926,992	609,116,213	4,517,988	5.06	95.55
14	125,194,864	116,442,268	607,970,623	4,531,884	4.85	93.01
15	103,494,974	99,239,967	543,851,152	3,998,763	5.25	95.89
16	98,319,150	92,840,807	534,474,134	3,928,889	5.44	94.43
17	95,272,651	89,677,039	468,395,835	3,456,144	4.92	94.13
18	90,772,031	85,715,767	456,115,712	3,373,974	5.02	94.43
19	61,342,430	56,286,798	291,811,698	2,148,157	4.75	91.76
X	166,650,296	148,955,095	469,399,660	3,543,869	2.82	89.38
Y	15,902,555	2,427,223	9,762,866	80,060	0.62	15.26

The CHORI-29 library covers 93.9% of the mouse genome at an average depth of 5.0-fold across the autosomes and 2.8-fold across the X chromosome. For further information regarding the data, please see legend for Table 2A.
